# Osimertinib in Early-Stage EGFR-Mutated Non-small Cell Lung Cancer: A Narrative Review of Its Impact on Survival, Safety, and Clinical Outcomes

**DOI:** 10.7759/cureus.72862

**Published:** 2024-11-01

**Authors:** Mohammed Khaleel Almadhoun, Salsapil Faris, Naeel Al-Far, Abdrhman Al-Hmarat

**Affiliations:** 1 Medicine, Al-Bashir Hospital, Amman, JOR; 2 Oncology, Al-Bashir Hospital, Amman, JOR; 3 Internal Medicine, Al-Bashir Hospital, Amman, JOR; 4 Neurology, Al-Bashir Hospital, Amman, JOR

**Keywords:** early-stage lung cancer, egfr mutations, lung cancer, osimertinib, treatment outcomes of osimertinib

## Abstract

Lung cancer remains a significant global health issue, with early detection playing a critical role in improving patient outcomes. The treatment landscape for advanced non-small cell lung cancer (NSCLC) has evolved substantially, especially with the introduction of targeted therapies such as osimertinib, a third-generation EGFR-tyrosine kinase inhibitor (TKI). This narrative review examines the impact of osimertinib on survival rates in early-stage lung cancer and underscores the importance of early diagnosis and treatment initiation. We explore the genetic underpinnings of NSCLC, focusing on the role of EGFR mutations, and discuss the prevalence of early-stage lung cancer and the urgent need for timely intervention.

Additionally, the review assesses osimertinib’s safety and efficacy in patients with early-stage EGFR-mutated lung cancer, drawing from a wide range of studies and clinical trials. Findings suggest that osimertinib has the potential to improve overall survival (OS) and progression-free survival (PFS) compared to traditional treatments such as chemotherapy, surgery, and first-generation EGFR-TKIs. The therapeutic profile of osimertinib is comprehensively analyzed, including its associated adverse events, clinical outcomes, and its impact on quality of life. We also address potential challenges with osimertinib, such as resistance mechanisms and the need for personalized treatment approaches. Despite these challenges, osimertinib emerges as a promising and well-tolerated treatment option for patients with EGFR mutations in early-stage lung cancer. The review highlights the importance of incorporating osimertinib into standard treatment regimens to improve patient outcomes and survival rates in early-stage lung cancer.

## Introduction and background

Lung cancer remains the leading cause of cancer-related deaths worldwide, accounting for approximately 1.6 million deaths annually [1]. Non-small cell lung cancer (NSCLC) is the most prevalent subtype, representing about 1.36 million (85%) of all lung cancer diagnoses. NSCLC includes several types of lung cancers, such as lung adenocarcinoma (LUAD), lung squamous cell carcinoma (LUSC), and large cell carcinoma, which are grouped together based on their histological, molecular, and clinical differences from small cell lung cancer (SCLC). The International Association for the Study of Lung Cancer (IASLC) classifies stages I, II, and IIIA as early-stage NSCLC [2].

Around 0.4 million (20-25%) of lung cancer patients are diagnosed at this early stage, and the standard treatment is radical surgical resection of the tumor [3]. However, recurrence rates remain high, ranging from 0.4 to 1.2 million (25-70%), likely due to microscopic metastases that are not eliminated during surgery. To reduce the risk of recurrence and improve survival, adjuvant chemotherapy or targeted therapy is typically recommended after surgery. Cisplatin-based chemotherapy is currently the first-line treatment for patients with resected NSCLC that has mutations in the EGFR [4,5].

While cisplatin-based chemotherapy has been the standard, its toxicity profile is less favorable compared to targeted therapies or immunotherapy. In a 2020 state-of-the-art review, researchers noted that, to date, molecularly targeted therapies had not shown significant OS benefits in early-stage NSCLC. However, numerous clinical trials have since been conducted to evaluate the efficacy and safety of targeted therapies in resected NSCLC. This review focuses on newer molecularly targeted treatment approaches, particularly the third-generation EGFR inhibitor, osimertinib. A comprehensive search was conducted using the MeSH terms "Carcinoma, Non-Small-Cell Lung" and "osimertinib." All relevant clinical trials and review articles on the use of osimertinib in early-stage or resectable NSCLC were included in this narrative review.

## Review

Diagnosis and staging of early-stage lung cancer

Lung cancer is most diagnosed in patients presenting with cough, fatigue, dyspnea, hemoptysis, and weight loss. Initially, these investigations included imaging studies such as chest X-ray, computed tomography (CT), and positron emission tomography (PET) scans. Histological examination of the biopsied tissue confirms the diagnosis and identifies the histological and molecular type of the tumor. Tissue approaches for histological evaluation include bronchoscopy, fine needle aspiration cytology (FNAC), and surgical resection [1,6]. A biopsy may not be performed before surgical resection in patients with a high suspicion of lung cancer in stage I or II. Liquid biopsy is a relatively newer technique to detect cancer biomarkers such as circulating tumor DNA (ctDNA) and circulating tumor cells (CTC) in plasma, serum, or urine. This technique has been used for the molecular analysis of tumor cells. Molecular analysis of a tumor is essential in choosing tailored therapy. Osimertinib is effective only against EGFR-mutant NSCLCs. ctDNA testing is highly specific (80-95%) and reasonably sensitive (60-85%) for identifying EGFR-activating mutations [6]. The Gaurdant360 cell line-derived xenograft (CDx) assay, an FDA-approved liquid biopsy, has also been used to detect EGFR mutant tumors. The clinical trial AURA2 used real-time PCR (rt-PCR) to detect EGFR T790M-resistance mutations in patients' plasma. Various committees have developed staging systems for NSCLC. The most commonly used staging system by clinicians is The TNM staging system for NSCLC, designed by the American Joint Committee (AJCC) on cancer [7].

The recent eighth edition of the AJCC NSCLC staging system revised the T categories to reflect the tumor size and extent more accurately. The tumor size thresholds were adjusted based on the prognostic analysis. Additionally, the eighth edition recognized sub-lobar resection as the first-line treatment for stage IA and tumors less than 2 cm in size. Stages I, II, and IIIA were classified as early-stage, resectable NSCLC by the AJCC and IASLC staging guidelines [7,8]. There is a localized tumor with no nodal metastasis in stages I and IIA. In stages IIB and IIIA, the malignant cells have spread to the ipsilateral hilar and mediastinal lymph nodes, respectively [2].

Treatment approaches for NSCLC

The treatment approaches for NSCLC depend upon its stage, histology, and tumor molecular characteristics. For stages I, II, and IIIA, surgical resection of the tumor is the standard of care [6]. For stages II-IIIA, adjuvant platinum-based chemotherapy is recommended postoperatively to decrease the risk of relapse [6,9]. Patients with stage IB NSCLC should be followed postoperatively, and a risk-benefit analysis should be done before starting chemotherapy [10]. Patients diagnosed with stage IIIB or above who have locally advanced disease are not candidates for surgical resection. Chemotherapy and targeted therapies are the first-line treatment approaches in these cases. Target therapy is based on the molecular characteristics of the tumor. TKIs have been approved for use in EGFR, a human gene that encodes a protein called B-Raf (BRAF), anaplastic lymphoma kinase (ALK), and RET genetic alterations. In case of no targetable alterations, PD-L1 inhibitors may be used for tumors expressing PD-L1 [6].

EGFR inhibitors

The first driver oncogene identified in NSCLC was EGFR mutation [9]. The development of tyrosine kinase inhibitors (TKI) against activating EGFR mutations has revolutionized the treatment of NSCLC. These drugs offered a better efficacy and safety profile compared to the standard platinum-based chemotherapy. EGFR, a tyrosine kinase receptor, functions by binding its ligands, transforming growth factor-alpha (TGFα) and epidermal growth factor (EGF). This binding leads to receptor dimerization. The dimer activates a series of intracellular signaling cascades, namely, the rat sarcoma-mitogen-activated protein kinase (ras-MAPK) pathway and the phosphoinositide 3-kinase-protein kinase B (P13K-AKT) pathway [11]. The activation of these pathways regulates cellular growth, division, and differentiation. EGFR activation also promotes cell survival by inhibiting apoptosis. In EGFR-mutated NSCLC, EGFR overexpression promotes cell proliferation, survival, invasion, and metastasis [12]. Inhibitors of this receptor were the first agents to be used as targeted therapy against cancerous cells. The first generation TKIs to be introduced were gefitinib and erlotinib [9,13]. They reversibly bind to the kinase domain of the EGFR and are competitive inhibitors of ATP [15]. Gefitinib and erlotinib had an overall response rate of 0.5-0.74 million (56% to 74%) in advanced EGFR-mutated NSCLC, and the median PFS was 10 to 14 months [14]. However, after a period of nine to 12 months, cancerous cells invariably develop resistance to the initial treatment. Threonine to methionine substitution at the 790 amino acid position (T790M) in the EFGR gene and met amplification are the two mechanisms implicated in the resistance to first-generation EGFR-TKIs [15]. Second-generation irreversible EGFR-TKIs, afatinib and dacomitinib, were introduced to overcome this resistance mechanism. In preclinical trials, they demonstrated response rates of less than 0.1 per million (10%) and were more toxic than the prototype first-generation TKI, gefitinib [9,14].

Understanding osimertinib

Osimertinib, a third-generation EGFR-TKI, is a mono-anilino-pyrimidine compound that irreversibly binds to the EGFR-kinase domain inhibiting its phosphorylation [9,15]. Osimertinib is also a potent T790M-mutated EGFR inhibitor, an acquired resistance in approximately 0.6 million (60%) of the patients previously treated with first- or second-generation EGFR-TKIs [9,16]. The replacement of threonine by methionine in the EGFR gene reduces the affinity of the (adenosine triphosphate) ATP-binding pocket of EGFR for first- and second-generation TKIs. Osimertinib, on the other hand, specifically binds to the kinase domain of the mutated EGFR, inhibiting ATP binding and autophosphorylation of the receptor. The downstream signaling pathways are blocked resultantly, and tumor growth is halted [16]. The drug has also shown efficacy against less common sensitizing mutations like exon 19 deletions or L858R mutation [17]. The first clinical trial in which osimertinib (known initially as AZD9291) was tested was a phase I dose escalation study conducted by Janne et al. [14]. This was the Phase I component of the AURA trial. The primary objective of this trial was to assess the safety and tolerability of osimertinib in patients with advanced NSCLC who had developed resistance to first-generation EGFR-TKIs due to the T790M mutation. The trial also aimed to establish the recommended dose for further studies. This study's response rate was 61%, and the median PFS was 9.6 months. In the pooled analysis of the subsequent phase II trials, the response rate of advanced T790M-positive NSCLC was 0.66 million (66%), and the median PFS duration was 11 months [18]. Based on these studies, osimertinib was Food and Drug Administration (FDA)-approved for advanced EGFR mutated and T790M positive NSCLC.

Impact of the early stage on survival

The American Cancer Society tracked the Survival, Epidemiology, and End Results (SEER) database to report five-year survival rates of patients diagnosed with NSCLC. The five-year survival rate was 65% in patients with localized disease, i.e., stage I and IIA. 37% of the patients with nodal metastasis, i.e., stage IIB and IIIA, were alive after five years. In comparison, only 0.09 million (9%) of the patients with advanced disease survived more than five years. The stage at the time of diagnosis is the most important prognostic factor for NSCLC patients. Early detection and prompt treatment are associated with a higher five-year survival rate. The presence of specific genetic mutations or molecular markers, such as EGFR or ALK mutations, can guide targeted therapy decisions and affect treatment response. The overall health and functional status of the patient, as well as their age and the presence of other comorbidities, can also affect the ability to tolerate treatments and contribute to survival outcomes. Multidisciplinary treatment approaches, including surgery, radiation therapy, and systemic therapies tailored to each patient's unique characteristics, are essential to optimize survival in early-stage lung cancer patients [19].

Introduction to osimertinib in the early stage

Osimertinib, as a standard therapy, has been approved by the FDA and the European Medicines Agency (EMA) for the treatment of resected early-stage NSCLC, thanks to the results of the double-blind phase III ADAURA trials, which evaluated the role of osimertinib in stages IB to IIIA EGFR-mutated early-stage NSCLC [20]. An unprecedented improvement in disease-free survival was (DFS) observed in the osimertinib arm across all predefined subgroups, including disease stages IB, II, and IIIA compared to placebo irrespective of the use of adjuvant chemotherapy or not. Out of a total of 682 patients undergoing randomization, 339 (49.7%) received osimertinib and 343 (50.3%) received a placebo with 22.5 months as the mean duration of treatment exposure [10].

The safety and efficacy of osimertinib as a neoadjuvant monotherapy or in combination with chemotherapy compared to chemotherapy alone in patients with resectable stage II-IIIB N2 EGFRm NSCLC, prior to surgery and adjuvant treatment, is being evaluated through the phase III NEOADAURA trial in which the primary objective is to assess the benefit and secondary objectives include event-free survival (EFS), efficacy assessments such as pCR, N2 to N0/N1 and N1 to N0 downstaging at the time of resection, DFS and OS.

Secondary endpoints include health-related quality of life (HRQoL), MPR in patients with or without detectable EGFRm at screening in ctDNA, and the concordance of EGFR mutation status between baseline tumor DNA and ctDNA samples, as well as between local and central tumor test results. Additionally, the pharmacokinetics of osimertinib and its metabolites, along with safety and tolerability, will also be assessed as secondary endpoints. Exploratory objectives include assessment of health resource use, tumor metabolism, and the association of biomarkers and minimal residual disease (MRD) with clinical response [21].

Osimertinib's efficacy in early stage

The percentage of patients who were alive and disease-free at 24 months was 89% (95% CI, 85 to 92) in the osimertinib group and 52% (95% CI, 46 to 58) in the placebo group (overall hazard ratio for disease recurrence or death, 0.20; 99.12% CI, 0.14 to 0.30; P<0.001) indicating significantly longer DFS among patients in the osimertinib arm compared to those in placebo arm. DFS of the patients with stage II to IIIA (470 in number), which was the primary endpoint of the trial, was 90% (95% CI, 84 to 93) in the Osimertinib group and 44% (95% CI, 37 to 51) in the placebo group (overall hazard ratio for disease recurrence or death, 0.17; 99.06% CI, 0.11 to 0.26; P<0.001) indicating 83% reduction in the risk of disease recurrence or death in the Osimertinib group [10].

A network meta-analysis of 10 trials involving 2707 patients with resected EGFRm + NSCLC evaluated the effect of osimertinib on DFS in comparison to gefitinib (HR 0.42, 95%CI 0.26-0.67) and erlotinib (HR0.4, 95%CI 0.24-0.66) demonstrating a consistent and more significant DFS benefit seen with osimertinib than that in other earlier generation EGFR-TKIs in patients with resected early-stage EGFRm + NSCLC [22]. Among the patients who received adjuvant chemotherapy, 89% (95% CI, 83 to 93) in the osimertinib group and 49% (95% CI, 41 to 56) in the placebo group were alive and disease-free at 24 months (overall hazard ratio for disease recurrence or death, 0.16; 95% CI, 0.10 to 0.26). Among the patients who did not receive adjuvant chemotherapy, 209 out of 235 (89%) (95% CI, 81 to 94) in the osimertinib group and 157 out of 271 (58%) (95% CI, 49 to 67) in the placebo group were alive and disease-free at 24 months (overall hazard ratio for disease recurrence or death, death was 0.23 (95% CI, 0.13 to 0.40) [10].

In the entire study population, the osimertinib group had a locoregional-only recurrence in 7% (23 out of 339 patients), while the placebo group had a higher rate of 18% (61 out of 343 patients). Distant recurrence, either alone or with locoregional recurrence, was observed in 4% (14 out of 339 patients) in the osimertinib group and in 96 out of 343 patients (28%) in the placebo group.

In a separate meta-analysis that included 1283 patients with resected EGFR-mutant NSCLC, it was found that the risk of CNS recurrence in those who received osimertinib was significantly lower (odds ratio of 0.11, 95% CI 0.04-0.32) compared to those who received gefitinib or erlotinib (odds ratio of 0.95, 95% CI 0.36-2.49) [23]. In a study evaluating the relationship between osimertinib concentration and clinical response in 25 Japanese patients with NSCLC, the optimal cut-off for plasma concentration of Osimertinib was set as 211 to categorize patients into high and low trough concentration groups. The median PFS calculated showed a significant difference between the two groups, 46.3 months in high concentration group and 16.8 months in low concentration group, whereas there was no significant difference in median OS [24].

Side effects and safety profile of osimertinib

**Figure 1:**
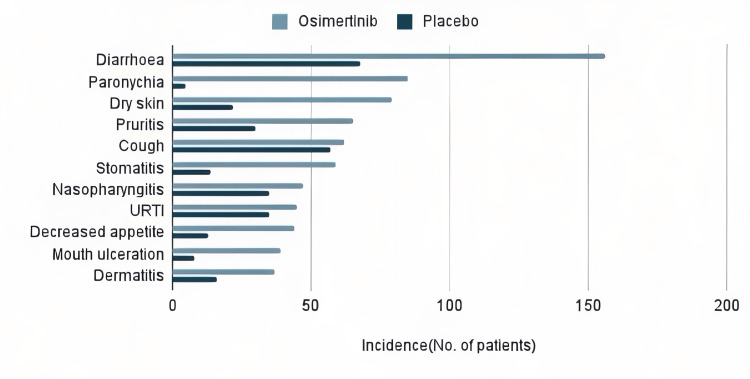
The figure illustrating the frequency of adverse events associated with osimertinib in patients AEs, adverse events

The secondary endpoints of the ADAURA trial included DFS in the overall population of patients with stage IB to IIIA disease, OS, HRQoL, and safety with HRQoL assessed by the SF-36 health survey version 2, third edition at baseline, week 12, week 24, and every 24 weeks until recurrence, treatment completion or discontinuation and safety assessments performed at baseline, week two, week four, week 12, and every 12 weeks until treatment completion or discontinuation [25]. Out of the total patients evaluated, 330 in osimertinib group and 339 in placebo group reported adverse events (AEs) out of which diarrhea (159 (47%)) was the most common AE with osimertinib followed by paronychia (92 (27%)), dry skin (84 (25%)), nausea (34 (10%)) and skin rash (33 (10%)), as illustrated in Figure 1 [10,25].

The percentages reported in the placebo group were as follows: diarrhea in 70 patients (20%), paronychia in five patients (1%), dry skin in 23 patients (7%), nausea in 20 patients (6%), and skin rash in 12 patients (3%). Serious adverse effects were reported in 68 patients (20%) among the osimertinib group and 47 patients (14%) in the placebo group with pneumonia being the most common, reported in five patients (1%) and four patients (1%) in the osimertinib and placebo groups, respectively [25]. ILD as an AE was the most common leading to discontinuation of osimertinib. Discontinuation was reported in 43 (13%) and nine (3%) patients in osimertinib and placebo groups, respectively; AEs resulting in death were rare in both the osimertinib and placebo groups, with fewer than 1% of patients affected in each group. In the osimertinib group, one patient experienced respiratory failure, while in the placebo group, two patients experienced pulmonary embolism and an unknown fatal event. These events were not considered to be causally related to the study treatment. In the mentioned Japanese study, decreased platelet count was the most common AE in both low trough and high trough concentration groups whereas paronychia was significantly higher in the high trough concentration group than the low trough concentration group. A dose reduction from the standard 80 mg dose was required in five patients from the low trough concentration group and two patients from the high trough concentration group [24].

Treatment monitoring and follow-up

According to AstraZeneca's guidelines from 2015, in the case of grade 3 AEs associated with osimertinib, its administration should be temporarily stopped for a period of up to three weeks. Afterward, treatment can be resumed either at the full dose of 80 mg daily or at a reduced dose of 40 mg daily, depending on the improvement of symptoms, which should be reduced to grade 0 to 2. However, if the rash does not adequately improve despite the interruption of EGFR-TKI therapy, osimertinib treatment should be permanently discontinued [26].

Cardiac events, like ejection fraction decrease, cardiac failure, pulmonary edema, and cardiomyopathy, were observed in 19 patients (6%) of the osimertinib group and nine patients (3%) of the placebo group, mostly being of grade 1 or 2 severity. There were five patients with grade 3 events, with four (1.2%) in the osimertinib group and one (0.3%) in the placebo group. The most common cardiac effect reported was ejection fraction decrease, with 15 (4.4%) cases in the osimertinib group and nine (2.6%) in the placebo group leading to treatment discontinuation (TTD) in two (0.6%) osimertinib group patients and three (0.9%) placebo group patients. QTc prolongation was observed in 30 patients (9%) of the osimertinib group and eight patients (2%) of the placebo group. The majority of cases were of grade 1 or 2 severity, with 26 patients (8%) in the osimertinib group and seven patients (2%) in the placebo group. There were five patients who experienced grade 3 events, with four patients (1%) in the osimertinib group and less than one patient (1%) in the placebo group. In total, five patients discontinued the study treatment due to QTc prolongation, with four patients (1%) in the osimertinib group and less than one patient (1%) in the placebo group. Although QT prolongation is rare, for osimertinib, it is advised to regularly perform an electrocardiogram (ECG) in patients at risk for QT prolongation [25,27,28]. Updated analyses on HRQoL revealed that most patients in both treatment groups experienced either stability or improvements in SF-36 Physical Component Summary (PCS) and Mental Component Summary (MCS) T-scores up to week 156 compared to their baseline scores. Additionally, there were no significant differences observed in the time to deterioration (TTD) of PCS or MCS between the osimertinib and placebo treatment groups [25,29].

The utilization of EGFR-TKI PET tracers has the potential to create predictive biomarkers, enabling the identification and monitoring of patients likely to respond positively to EGFR-TKI therapies. Currently, these biomarkers are undergoing validation, with continuous iterations involving clinical and analytical enhancements. Recent advancements, such as the availability of large-FoV total body PET systems and improved data processing algorithms, can enhance the validation process of EGFR-TKI PET biomarkers. However, further evidence is required to establish their qualification as reliable predictive and monitoring biomarkers for drug development and routine clinical application.

Resistance to osimertinib 

Osimertinib is being used in more clinical settings, but resistance to it is also rising (Table 1). The mechanism of resistance of osimertinib can be divided into two types: (a) intrinsic resistance and (b) extrinsic resistance.

**Table 1:** Osimertinib combination therapy for resistance MTD, maximum tolerated dose; ORR, objective response rate; PFS, progression-free survival; AEs, adverse events; DLTs, dose-limiting toxicities; RP2D, recommended phase 2 dose; DFS, disease-free survival; CT, chemotherapy; MP, major pathological

NCT number	Phase	Number of patients	Study status	Intervention	Primary outcome	Estimated study completion date
NCT03567642	I	20	Recruiting	Osimertinib + cisplatin/carboplatin + etoposide	MTD (maximum tolerated dose)	June 2024
NCT03122717	I/II	48	Active, not recruiting	Osimertinib + gefitinib	Number of patients completing 6 cycles	April 2025
NCT03392246	II	25	Active, not recruiting	Osimertinib + selumetinib (MET inhibitor)	ORR	June 30, 2026
NCT03810807	I	22	Active, not recruiting	Osimertinib + dacomitinib	MTD	January 2024
NCT02954253	I	10	Active, not recruiting	Osimertinib + masitinib	MTD	June 2023
NCT02971501	I	112	Active, not recruiting	Osimertinib + bevacizumab	PFS	July 1, 2024
NCT03909334	II	150	Recruiting	Osimertinib, ramucirumab	PFS	June 2024

Intrinsic resistance to osimertinib

**Figure 2:**
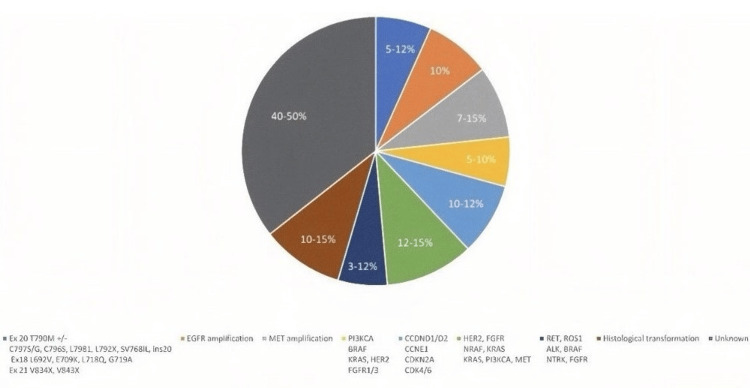
Overview of the main resistance mechanisms to osimertinib's first-line therapy, including their frequency and types

Despite the fact that the majority of research only considers osimertinib resistance during second-line therapy, certain literature data reports intrinsic resistance when it is used as a first-line treatment as shown in Figure 2 [30]. It has been discovered that third-generation EGFR-TKIs such as osimertinib are less sensitive in patients with HER2 and MET gene mutations [31]. Additionally, KRAS G12D mutation and PTEN loss were found in NSCLC patients who had intrinsic osimertinib resistance [32]. Patients with NSCLC who had the EGFR mutation and CDCP1 or AXL RNA overexpression reported having the poorest response to third-generation EGFR-TKIs (Table 2) [32].

**Table 2:** Overcoming resistance to osimertinib MTD, maximum tolerated dose; ORR, objective response rate; PFS, progression-free survival; AEs, adverse events; DLTs, dose-limiting toxicities; RP2D, recommended phase 2 dose; DFS, disease-free survival; CT, chemotherapy; MP, major pathological

NCT number	Phase	Number of patients	Study status	Intervention	Primary outcome	Estimated study completion date
NCT03891615	I	30	Recruiting	Osimertinib + niraparib (PARP inhibitor)	Maximum tolerated dose of niraparib	April 30, 2024
NCT03944772	I	250	Recruiting	Osimertinib + savolitinib (MET inhibitor)	ORR	November 28, 2025
NCT03523698	II	100	Unknown	Osimertinib + aspirin	ORR	December 31, 2022
NCT03778229	II	360	Recruiting	Osimertinib + savolitinib	ORR	February 28, 2025
NCT04001777	I	80	Recruiting	APG-1252 + osimertinib	MTD, RP2D	June 2025

Extrinsic resistance to osimertinib 

**Figure 3:**
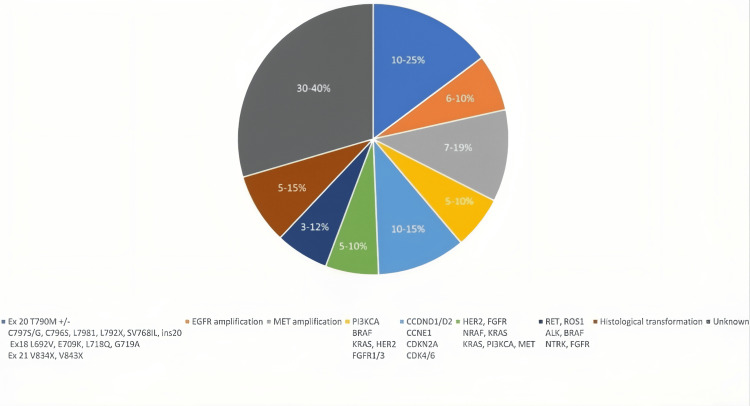
Overview of the main resistance mechanisms to osimertinib's later-line therapy, including their frequency and types

Depending on the EGFR-TKI used and the course of treatment, the frequency of EGFR-dependent acquired resistance varies, which is summarized in Figure 3 (Table 3). Tertiary mutation in the EGFR residue C797 in exon 20 in the ATP-binding region is one of the important causes of osimertinib resistance [33]. Osimertinib overcomes T790M resistance by creating a covalent link with the residue C797 in the ATP pocket; as a result, the protein change brought on by a mutation in the C797 residue prevents osimertinib from binding to the mutant EGFR, making it ineffective [33].

**Table 3:** Overcoming resistance to prior EGFR-TKI MTD, maximum tolerated dose; ORR, objective response rate; PFS, progression-free survival; AEs, adverse events; DLTs, dose-limiting toxicities; RP2D, recommended phase 2 dose; DFS, disease-free survival; CT, chemotherapy; MP, major pathological; TKI, tyrosine kinase inhibitor

NCT number	Phase	Number of patients	Study status	Intervention	Primary outcome	Estimated study completion date
NCT02496663	I	138	Active, not recruiting	Osimertinib + necitumumab	MTD	June 30, 2024
NCT02503722	I	36	Active, not recruiting	Osimertinib + sapanisertib (mTOR inhibitor)	DLT, MTD	August 31, 2023
NCT02520778	I	50	Active, not recruiting	Osimertinib + navitoclax (Bcl-2 inhibitor)	Feasibility, AEs	July 30, 2023

**Figure 4:**
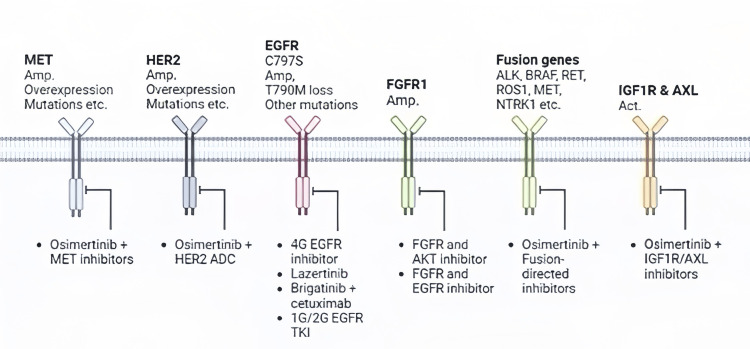
A brief summary of osimertinib resistance mechanisms along with suitable therapies Information has been adapted with permission [35-38].

Patients with osimertinib resistance have also been shown to have rare EGFR mutations, such as G796R, G796S, and G796D mutations along with L792 exon 20 mutations, such as L792H, L792Y, and L792F mutations [34,35]. The L718Q, L718V, and L798I mutations are another mechanism of resistance in exon 18, which affects the ATP-binding site of the EGFR kinase domain, preventing the binding of osimertinib [35,36]. Some other mutations including G719A, G724S, SV768IL (S768I + V769L), G724S, L718Q, V834L, and L718V have also been identified [36-38]. Numerous therapies have been proposed for the treatment of osimertinib-resistant lung cancer as demonstrated in Figure 4.

For patients with T790M/cis-C797S EGFR mutations, brigatinib, a dual ALK and EGFR inhibitor, in combination with cetuximab (anti-EGFR antibody) led to an objective response rate (ORR) of 60% and a median PFS of 14 [39]. Another combination of EGFR-TKIs with anti-VEGFR might be a promising therapy [39]. Numerous EGFR allosteric inhibitors of the next generation have been developed. They are found to be useful against the C797S-mediated resistance mechanism by attaching to the EGFR at a location other than the tyrosine kinase domain [40]. EAI045, JBJ-04-125-02, CH7233163, and BLU-945 are some of them [41-43].

Despite having useful effects, most of them have not been tested clinically. Furthermore, certain MET inhibitors in combination with third-generation EGFR-TKI have been tested for osimertinib resistance due to MET amplification. These include savolitinib, capmatinib, and tepotinib [44-46]. Some studies also mention EGFR-TKIs plus anti-EGFR antibodies, osimertinib plus CDK4/6 inhibitors, and third-generation EGFR-TKI plus Bcl-2 Inhibitors for osimertinib resistance. Immunotherapy is another treatment that is being introduced but it has lower sensitivity to anti-PD-1/PD-L1 treatment [47].

However, platinum-based chemotherapy is the best course of action, particularly in cases of SCLC transformation because 30-50% of the causes of osimertinib resistance are still unclear, and rebiopsies frequently fail to reveal any targetable alterations [48]. For patients with SCLC who had undergone a transformation, a retrospective analysis revealed significant activity of platinum-etoposide (ORR of 54%, mPFS of 3.4 months, and OS of 10.9 months) [49]. Another retrospective analysis revealed that chemotherapy improves survival (25.0 versus 11.8 months) in advanced NSCLC patients who progressed while receiving osimertinib first-line treatment without a molecular target over a nonchemotherapy approach [48]. However, further clinical evidence is needed to prove its effectiveness.

Multidisciplinary approach to lung cancer

The complexity of the patient population and the disease's underlying biological variety make managing lung cancer difficult; hence, close cooperation between professionals with multiple specialties is necessary for treatment alternatives. According to the European Society for Medical Oncology (ESMO), multidisciplinary care delivery can be done in a number of different ways [50]. One approach is a regular multidisciplinary meeting (MDM), case conference, or tumor board where treating experts attend a meeting to present and discuss the best possible choices for patient treatment [51].

Another is a multidisciplinary clinic (MDC), a centralized lung cancer clinic where patients can consult with the necessary specialist. According to the National Comprehensive Cancer Network (NCCN) for patients with metastatic NSCLC first-line osimertinib (preferred), erlotinib, afatinib, gefitinib, and dacomitinib are recommended, with second-line alternatives being considered depending on the patient's disease features and/or 1L therapy history [52]. Similarly, ESMO recommends osimertinib (preferred), erlotinib, gefitinib, afatinib, dacomitinib, as well as combinations of erlotinib with bevacizumab or ramucirumab, and the addition of first-line carboplatin and pemetrexed to gefitinib for patients with sensitizing EGFR mutations in mNSCLC [53].

These guidelines are supported by multiple studies including the FLAURA trial, which has established osimertinib as the standard of care in patients with mNSCLC [53]. AURA3 and TREM are other trials that confirm the efficacy of osimertinib as a second-line treatment for patients with T790M [54,55]. Unfortunately, patients receiving osimertinib eventually develop resistance, rendering the therapy ineffective [34]. To enhance survival and treatment outcomes, new approaches including multidisciplinary care, a combination of EGFR-TKI, and fourth-generation EGFRI-TKI are found to be efficient.

Treatment results are also significantly influenced by the patient's cooperation and awareness of the process. The Institute of Medicine has emphasized collaborative decision-making and consideration of patient preferences in order to improve the quality of cancer care [56]. Research reveals that patients who are younger and less educated are less likely to participate in decision-making; moreover, patients who actively engage in treatment choices are found to be more satisfied [57,58]. Multidisciplinary care is another strategy, which has increased patients’ satisfaction and, ultimately, treatment outcomes as it provides professionals belonging to different medical fields working synergistically [51].

Patient selection and eligibility for osimertinib 

The main consideration for choosing patients to get osimertinib is if their tumors have the EGFR T790M mutation, which acts as a mechanism of resistance to their prior EGFR-TKI treatment [59]. Hence, to test for this mutation, initial EGFR testing utilizing plasma samples is advised by the National Comprehensive Care Network (NCCN) [60]. Further tissue biopsy due to the inadequate sensitivity of plasma study is needed by some individuals [61]. In addition, various studies reported finding EGFR T790M mutations in the second or third rebiopsies even if the initial rebiopsy did not reveal them. In a recent study by Seto et al., EGFR T790M mutations were found in 5.7% of patients in the second rebiopsy and in 12.5% of patients in the third rebiopsy, even though the initial rebiopsy did not find the EGFR T790M mutation [61]. Before selecting Osimertinib, various additional considerations, such as tumor burden, should be made. FLOWER study found that individuals with brain metastases, presence of symptoms, and at least three metastatic sites at diagnosis had poorer treatment outcomes, suggesting tumor burden as a negative prognostic indicator [62]. Besides that, aged patients and individuals with comorbidities and less prevalent EGFR mutations did not show a difference in PFS, TTD, or OS, indicating successful administration of osimertinib in these subpopulations as well.

Future perspectives and research directions 

Osimertinib's effectiveness in treating NSCLC has been studied in several studies. In the phase III study ADAURA, 682 stage IB-IIIA EGFRm NSCLC patients were randomized to receive Osimertinib 80 mg once daily or a placebo for three years to access DFS benefit (stage II-IIIA DFS HR, 0.23; 95% CI: 0.18-0.30; stage IB-IIIA DFS HR, 0.27; 95% CI: 0.21-0.34) [63]. In the phase 2b trial (ChiCTR1800016948), patients with a measurable stage IIA-IIIB (T3-4 N2) lung adenocarcinoma and EGFR exon 19 and/or 21 mutations were treated with osimertinib 80 mg orally once per day for six weeks, followed by surgical resection. The primary endpoint was the ORR (71.1 % (27/38) (95% CI: 55.2-83.0) in 38 patients who completed the six-week osimertinib treatment. Thirty-two patients underwent surgery, and 30 (93.8 %) underwent successful R0 resection [64]. 

In ASTRIS, the biggest real-world trial of second- or later-line osimertinib in advanced or metastatic EGFR T790M NSCLC, patients were given 80 mg of osimertinib once daily if they already had undergone EGFR-TKI therapy for their malignancy [65]. The primary endpoint was median OS: 22.8 months (21.6-23.8), and the secondary endpoints were median PFS: 11.1 months (11.0-12.0), median TTD: 13.5 months (12.6-13.9), and response rate: 57.3% (55.5-59.2).

OSIREX was another trial in which patients with advanced or metastatic NSCLC EGFR-T790M mutation-positive were given osimertinib to evaluate median PFS, which was found to be 9.4 months [66]. ADAURA2 is an ongoing trial evaluating DFS in participants with EGFRm stage IA2-IA3 NSCLC following complete tumor resection by administering 80 mg osimertinib, once daily [67]. A phase II trial is being carried out to assess the effectiveness of osimertinib in treating patients with stage I-IIIA EGFR-mutant NSCLC prior to surgery [68]. The primary outcome of this study is the major pathological response rate (MPR).

Several studies have used osimertinib in combination with other drugs. In the phase Ib study, osimertinib is combined with either savolitinib (AZD6094, MET inhibitor), selumetinib (MEK inhibitor), or durvalumab (anti-PDL1 antibody) [69]. Another phase Ib study with osimertinib and navitoclax is being conducted in patients who are on an anti-EGFR-TKI and have disease progression [70]. In a different trial, patients with advanced EGFR-mutated NSCLC are treated with osimertinib and sapanisertinib (INK128), a powerful mTORC1/2 inhibitor [71]. For patients who progressed on prior first-line TKI treatment, a phase I dose-escalation trial of osimertinib coupled with necitumumab, a humanized EGFR monoclonal antibody, has been conducted [72]. NeoADAURA is examining the advantages of neoadjuvant osimertinib over conventional chemotherapy when used both in conjunction with chemotherapy for three cycles and on its own for at least nine weeks [73].

## Conclusions

In summary, this review highlights the growing role of osimertinib in the treatment of early-stage EGFR-mutated NSCLC. The available evidence from clinical trials suggests that osimertinib offers significant improvements in both OS and PFS compared to conventional treatments, such as chemotherapy or earlier-generation EGFR inhibitors. Its targeted mechanism of action, coupled with a favorable safety profile, positions osimertinib as a valuable option for patients following surgical resection. However, the potential for resistance mechanisms, along with the need for individualized treatment approaches, remains an area for ongoing research.

Despite the challenges, osimertinib has demonstrated a well-tolerated and effective option for many patients with early-stage EGFR-mutated lung cancer. Incorporating this drug into standard post-surgical treatment regimens could lead to better long-term outcomes, reducing the risk of recurrence and improving patient survival rates. Continued research and clinical trials are necessary to fully understand its long-term efficacy, manage potential resistance, and refine treatment protocols for optimal use. Ultimately, osimertinib represents a promising advancement in the fight against lung cancer, offering hope for improved patient care and survival.
